# TRAIL-Dependent Resolution of Pulmonary Fibrosis

**DOI:** 10.1155/2018/7934362

**Published:** 2018-01-24

**Authors:** David M. Habiel, Ana Paula Moreira, Ugur B. Ismailoglu, Michael P. Dunleavy, Karen A. Cavassani, Nico van Rooijen, Ana Lucia Coelho, Cory M. Hogaboam

**Affiliations:** ^1^Women's Guild Lung Institute, Department of Medicine, Cedars-Sinai Medical Center, Los Angeles, CA 90048, USA; ^2^Immunology Group, Department of Pathology, University of Michigan Medical School, Ann Arbor, MI 48103, USA; ^3^Urologic Oncology Program and Uro-Oncology Research Laboratories, Samuel Oschin Comprehensive Cancer Institute, Cedars-Sinai Medical Center, Los Angeles, CA 90048, USA; ^4^Department of Molecular Cell Biology, Faculty of Medicine, Vrije Universiteit, Vrije Universiteit Medisch Centrum, 1081 BT Amsterdam, Netherlands

## Abstract

Idiopathic pulmonary fibrosis (IPF) is the most common form of interstitial lung disease characterized by the persistence of activated myofibroblasts resulting in excessive deposition of extracellular matrix proteins and profound tissue remodeling. In the present study, the expression of tumor necrosis factor- (TNF-) related apoptosis-inducing ligand (TRAIL) was key to the resolution of bleomycin-induced pulmonary fibrosis. Both *in vivo* and *in vitro* studies demonstrated that Gr-1^+^TRAIL^+^ bone marrow-derived myeloid cells blocked the activation of lung myofibroblasts. Although soluble TRAIL was increased in plasma from IPF patients, the presence of TRAIL^+^ myeloid cells was markedly reduced in IPF lung biopsies, and primary lung fibroblasts from this patient group expressed little of the TRAIL receptor-2 (DR5) when compared with appropriate normal samples. IL-13 was a potent inhibitor of DR5 expression in normal fibroblasts. Together, these results identified TRAIL^+^ myeloid cells as a critical mechanism in the resolution of pulmonary fibrosis, and strategies directed at promoting its function might have therapeutic potential in IPF.

## 1. Introduction

IPF is the most common clinical form of interstitial lung disease with a prognosis of median survival at 3–5 years after diagnosis and no effective pharmacological intervention [1–3]. IPF is characterized histologically by the presence of fibroblastic foci, which are believed to be the site of active tissue remodeling and deposition of extracellular matrix proteins due to presence of activated fibroblasts or myofibroblasts. The fibrotic triggers in IPF are unknown but it is speculated that a persistent injury to the lung leads to the death of alveolar epithelial cells and subsequent aberrant repair mechanisms ablate the alveolus [4–11]. A key feature associated with the aberrant repair process in fibrotic interstitial lung disease is the persistence of activated myofibroblasts, which are resistant to and/or lack components necessary to respond to proapoptotic signals [9–14].

TRAIL deficiency has been previously reported to exacerbate bleomycin-induced lung fibrosis in mice [15] but the manner in which TRAIL promoted the resolution of pulmonary fibrosis is presently unknown. TRAIL is a potent inducer of apoptotic cell death and binds to various cells via a number of death receptors of which there are five in humans: TRAIL-R1 (DR4), TRAIL-R2 (DR5), TRAIL-R3, TRAIL-4, and osteoprogenin (OPG); and four in mice: mTRAIL-R2 (mDR5), mDcTRAIL-R1, mDcTRAIL-R2, and OPG. DR4, DR5, and mDR5 contain a complete cytoplasmic death domain and can activate both the intrinsic and extrinsic apoptotic death pathways in response to the binding of TRAIL (reviewed in [16]). However, TRAIL-R3, TRAIL-R4, mDcTRAIL-R1, and OPG lack a complete death domain and are considered to be decoy receptors for TRAIL. TRAIL-based therapies have been extensively studied in the context of cancer but have yielded limited clinical success due to the variable sensitivity of cancer cells to this proapoptotic factor. One strategy that has been reported to enhance TRAIL susceptibility in cancer involves altering the expression of this ligand on bone marrow-derived mesenchymal cells [17]. These cells home to tumor sites and because of their regenerative capacity persist at these sites. Indeed, the bone marrow is an important source of cells of both mesenchymal and hematopoietic origin which protect, regenerate, and restore the lung following fibrosis [18–25]. The latter group of hematopoietic cells includes myeloid cells with potent immune regulatory properties, which are characterized by the concomitant surface expression of CD11b and Gr-1 in mice [26–31].

Herein, we employed clodronate liposome (Clod Lipo) and anti-Gr-1 mAb strategies to modulate the presence and function of Gr-1^+^ bone marrow-derived myeloid populations in bleomycin-challenged mice. Anti-Gr-1 mAb treatment has been shown to deplete this population, whereas clodronate liposomes have been shown to mobilize the release of bone marrow myeloid and other cell types [32, 33]. We observed that the depletion of Gr-1^+^ myeloid cells prevented the resolution of bleomycin-induced pulmonary fibrosis, whereas their mobilization from the bone marrow or the adoptive transfer of isolated Gr-1^+^ myeloid cells either prior to or after bleomycin challenge enhanced the rate of fibrotic resolution in this model via the TRAIL-dependent apoptosis of myofibroblasts. Clinically, IPF patients expressed elevated levels of circulating TRAIL but tissue biopsies from IPF patients exhibited few TRAIL^+^ myeloid cells and lower DR5 expression compared with normal samples. Markedly less DR5 expression was observed in IPF human fibroblasts as compared to normal fibroblasts, where the expression of this receptor was inhibited by IL-13. Thus, these data show that the presence of a TRAIL^+^ bone marrow-derived myeloid cell population in the lung exerts potent antifibrotic effects via their promotion of myofibroblast apoptosis. The promotion of TRAIL-dependent proapoptotic activity toward human fibroblasts might provide a much-needed therapeutic option for IPF.

## 2. Materials and Methods

### 2.1. Bleomycin-Induced Pulmonary Fibrosis

Male, C57BL/6 mice (6–8 weeks of age) were purchased from Taconic Farms Inc. (Hudson, NY) and maintained under specific pathogen-free conditions at the University of Michigan Medical School. The University of Michigan Committee on the Use and Care of Animals approved all protocols used in this study. Mice were anesthetized with ketamine/xylazine (100 mg/kg) and each mouse received 0.05 U of bleomycin sulfate (Bristol-Meyers Pharmaceuticals) dissolved in 50 *μ*l of PBS via oropharyngeal instillation. Groups of bleomycin-challenged mice (*n* = 10/group) received either anti-Gr-1 mAb (100 *μ*g/injection; RB6-8C5 (anti-Ly6G); eBioscience) or an isotype control (eBioscience) via an i.p. injection on day 21, and lungs were evaluated at day 42 after bleomycin challenge. To promote the release of myeloid cells from the bone marrow, macrophages were depleted using clodronate-encapsulated liposomes (purchased from http://clodronateliposomes.com and supplied as a 5 mg/ml solution). To achieve a full macrophage depletion, a dose of 0.1 ml/10 g is recommended by the manufacturer (http://clodronateliposomes.com). However, in our studies, 0.1 ml/20 g was utilized, which was effective in mobilizing Gr-1^+^ cells in the treated animals with no evidence of clodronate-induced toxicity. Briefly, groups of mice (*n* = 5–10/group) were treated with 0.1 ml of i.p. injected clodronate-encapsulated liposomes (abbreviated Clod Lipo) either 1 day prior to or at day 14 after bleomycin challenge. The same volume of PBS-encapsulated empty liposomes (abbreviated Lipo) was used as control one day prior to bleomycin. The liposome-treated groups of mice were analyzed at days 5, 14, and/or 21 after the bleomycin challenge.

### 2.2. Collection and Adoptive Transfer of Lung Gr-1^+^ Myeloid Cells

Mice received clodronate-encapsulated liposomes one day prior to bleomycin treatment (i.e., D−1). Lung Gr-1^+^ myeloid cells were purified from these mice at day 5 after bleomycin by magnetic-activated cell sorting (MACS; Miltenyi Biotechnologies) via the use of an anti-Gr-1 mAb. Groups of mice (*n* = 10/group) either at day 5 or 14 after bleomycin challenge received MACS-purified Gr-1^+^ myeloid cells (1 × 10^5^ cells in 50 *μ*l of PBS) via the oropharyngeal route under anesthesia. Both groups were analyzed at day 21 after bleomycin.

### 2.3. Coculture of Pulmonary Fibroblasts with Purified Gr-1^+^ Myeloid Cells

Pulmonary fibroblasts were isolated from naïve mice as previously described in detail [34] and plated at 5 × 10^5^ cells/well in a 6-well tissue culture plate. Cocultures were created with the addition of MACS-isolated lung Gr-1^+^ myeloid cells (1 × 10^5^ cells/well) to the cultured fibroblasts for 24 h. Cell-free supernatants were collected to determine protein levels, and the remaining adherent cells in these cocultures were subjected to RNA isolation for quantitative PCR. Other cocultures were subjected to confocal microscopy to visualize collagen expression by the adherent fibroblasts. Briefly, adherent fibroblasts were fixed and permeabilized and incubated with an anti-collagen-1 mAb (Abcam) for 24 h followed by Alexa flour 594-conjugated secondary antibody (Life Technologies). Each slide was then treated with Prolog Gold Anti-Fade containing DAPI (Life Technologies), coverslips were mounted, and images were analyzed with a Zeiss LSM510 confocal microscope with C-Apochromatic 40x objective lens (Carl Zeiss). Images were assembled using Adobe Photoshop CS2 for Mac. In other experiments, cultured mouse fibroblasts were exposed to increasing concentrations of sTRAIL for 24 h and RNA was then isolated for quantitative PCR analysis.

### 2.4. Histological and Cellular Analysis

Formalin-fixed and paraffin-embedded lung sections were stained with Masson trichrome to detect collagen deposition. Cytospin samples of bronchoalveolar lavage samples were prepared, fixed, and stained using a Quick-Diff staining kit.

### 2.5. RNA Isolation and Real-Time TaqMan PCR

Total RNA was prepared from lung samples using TRIzol reagent according to the manufacturer's directions (Life Technologies). RNA was reverse-transcribed into cDNA using M-MLV reverse transcriptase (Life Technologies). GAPDH was used as an internal control. Real-time quantitative PCR was carried out using a TaqMan 7500 sequence detection system (Applied Biosystems). Measurements were standardized to GAPDH levels and data were presented as fold changes from the naïve group.

### 2.6. ELISA

IL-10, IL-2, IL-13, IL-4, CCL22, CCL17, TGF-*β*, IFN-*γ*, and TRAIL levels were determined in whole lung homogenates using a standardized sandwich ELISA technique (R&D Systems). Recombinant murine proteins were used to generate standard curves (R&D Systems). Results were normalized to total protein amount measured by Bradford assay and the limit of detection for each protein was consistently above 50 pg/ml.

### 2.7. Flow Cytometry

Lung tissues were enzymatically digested and stained with intracellular or surface antibodies (BD Pharmingen) as previously described in detail [35]. Fixed cell suspensions were subjected to multicolor flow cytometry (Cytomics FC-500; Beckman Coulter). Data were analyzed with FlowJo software (TreeStar).

### 2.8. Human Samples

Plasma, lung biopsy, and primary fibroblast lines were obtained from nondiseased and IPF patients and analyzed using routine immunohistochemical techniques, ELISA, and quantitative PCR as described in detail elsewhere [36, 37]. Approval to use these blood, tissue, and cell samples was obtained from an institutional review board at the University of Michigan Medical School. Informed consent was obtained from all patients prior to inclusion in the studies described herein. All the studies were performed in accordance with the relevant guidelines and regulations.

### 2.9. Statistical Analysis

All results are expressed as mean ± SEM. Student's *t*-test or one-way ANOVA statistical analysis was used to detect differences. *P* value ≤ 0.05 was considered to be significant.

## 3. Results

### 3.1. Systemic Clodronate Liposome Treatment Promotes the Accumulation of CD11b^+^Gr-1^+^ Myeloid Cells in the Fibrotic Lung and Is Associated with Reduced Lung Fibrosis

**Figure 1:**
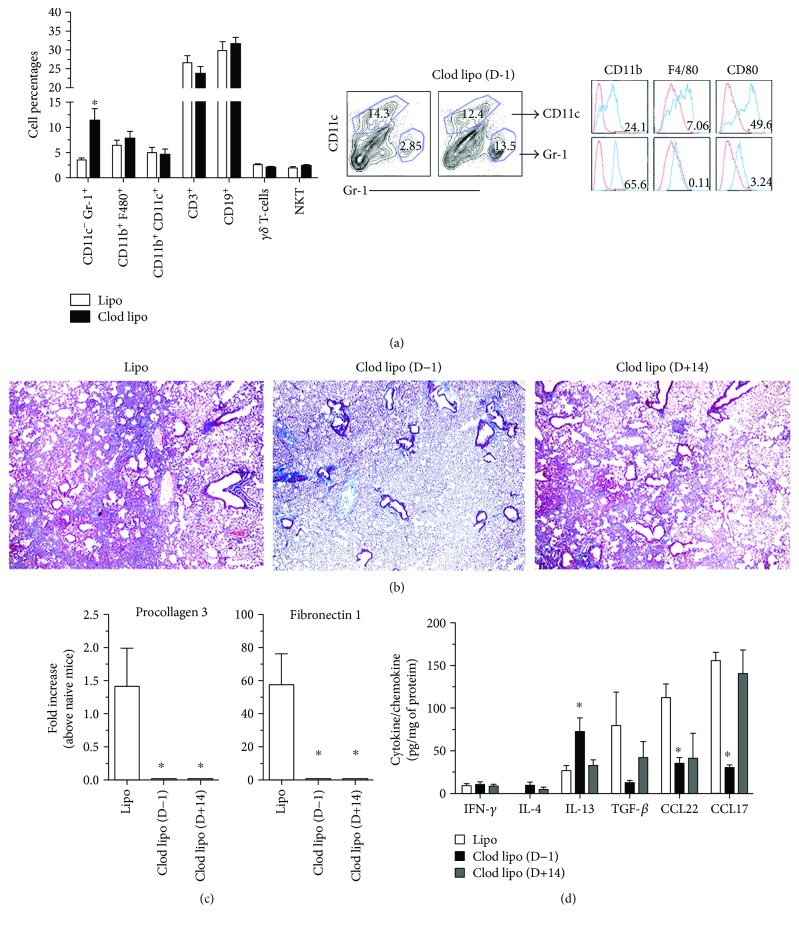
Bone marrow mobilization using systemically administered clodronate liposomes increased the accumulation of Gr-1^+^ myeloid cells in the lung and is associated with reduced pulmonary fibrosis. (a) Mice either received an intraperitoneal injection of liposomes containing clodronate (Clod Lipo) or empty liposomes (Lipo) at 1 day prior to (D−1) an intrapulmonary bleomycin challenge. All mice were subsequently sacrificed at day 5 after bleomycin challenge, and lungs from D−1 Clod Lipo- or Lipo-treated mice were digested to generate a cellular suspension, and cells were stained with monoclonal antibodies directed against Gr-1, CD11b, CD11c, F4/80, CD3, CD19, *γδ* TCR, or NK 1.1 and analyzed by flow cytometry. (b) Mice either received an intraperitoneal injection of liposomes containing clodronate (Clod Lipo) either 1 day prior to (D−1) or at 14 days after (D+14) or empty liposomes (Lipo) one day prior to intrapulmonary bleomycin challenge. All mice were subsequently sacrificed at day 21 after bleomycin challenge and their lungs were isolated for analysis. Whole lungs were fixed, paraffin-embedded, sectioned, and Masson's trichrome-stained to visualize ECM. Shown are representative images taken at 200x magnification. (c) Quantitative PCR analysis of procollagen 3 and fibronectin 1 in whole lung samples. (d) ELISA analysis of whole lung levels of IFN-*γ*, IL-4, IL-13, TGF-*β*, CCL22, and CCL17. Data are mean ± SEM, *n* = 5–10/group, ^∗^ *P* ≤ 0.05 compared with Lipo-alone group.

Clod Lipo has been previously utilized to mobilize bone marrow-derived cells into circulation; however, the effect of such mobilization during an acute pulmonary inflammatory response elicited by intratracheal bleomycin has not been previously reported. Systemic administration of Clod Lipo was employed to mobilize bone marrow cells one day prior to (i.e., D−1) bleomycin challenge, and whole lung was analyzed 5 days after bleomycin administration. As a control for any nonspecific effects of the liposomes, mice in the control group were injected with empty liposomes (i.e., Lipo) at the same time. Mice that received Clod Lipo exhibited a significantly augmented percentage (i.e., from 2.9% to 13.5%) of CD11c^−^Gr-1^+^ myeloid cells in lung samples removed at day 5 after bleomycin challenge compared with the Lipo-treated group (Figure 1(a)). Interestingly, there was no difference in the number of macrophages (CD11b^+^F4/80^+^), dendritic cells (CD11b^+^CD11c^+^), CD3^+^ T-cells, CD19^+^ B-cells, *γδ* T-cells, or NKT cells in the fibrotic lungs of the two treatment groups (i.e., Lipo versus Clod Lipo) (Figure 1(a)). We also observed that approximately 65% of CD11c^−^Gr-1^+^ myeloid cells from Lipo or Clod Lipo treatment also expressed CD11b, while only 0.1% and 3.2% of these cells expressed F4/80 and CD80, respectively.

To investigate the role of Gr-1^+^ myeloid cells in lung fibrosis, groups of mice were treated with Clod Lipo at D−1 or 14 days after (i.e., D+14) bleomycin challenge and whole lung samples were analyzed at day 21 after bleomycin. Clod Lipo treatment at D−1 markedly reduced the amount of histologically apparent (i.e., trichrome staining) lung fibrosis when compared to Lipo-treated (D−1) mice at day 21 after bleomycin (Figure 1(b)). When Clod Lipo was administered at D+14, lung fibrosis was also reduced as compared to Lipo-treated (D−1) mice at day 21 after bleomycin, but the trichrome staining in this group was greater than that observed in the D−1 Clod Lipo-treated group (Figure 1(b)). Nevertheless, both Clod Lipo-treated groups had significantly reduced transcript expression of procollagen 3 and fibronectin 1 in lung samples analyzed at day 21 after bleomycin (Figure 1(c)). Whole lung cytokine analysis demonstrated that there were no differences in IFN-*γ* and IL-4 and there were lower levels of TGF-*β*, CCL22, and CCL17 but higher (*P* ≤ 0.05) levels of IL-13 in the D−1 Clod Lipo-treated group compared with the Lipo-treated group (Figure 1(d)). Further ELISA analysis of whole lung samples from D+14 group demonstrated that there were no differences in IFN-*γ*, IL-4, IL-13, TGF-*β*, or CCL17 but CCL22 levels were lower in this group compared with the Lipo-treated group (Figure 1(d)). Together, these data suggested that mobilizing the release of Gr-1^+^ bone marrow myeloid cells using Clod Lipo markedly attenuated bleomycin-induced pulmonary fibrosis.

### 3.2. Gr-1^+^ Myeloid Cells Are Recruited to Fibrotic Lungs during Resolution Phase of Bleomycin-Induced Fibrosis

**Figure 2:**
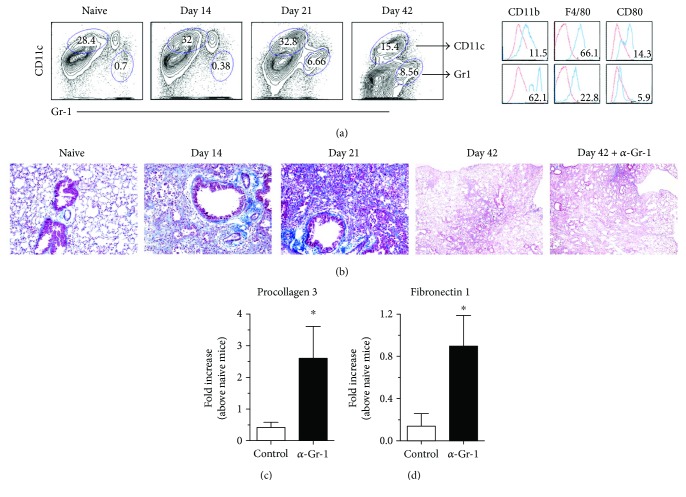
Gr-1^+^ myeloid cells were recruited in a temporal manner to the lung and their depletion exacerbated bleomycin-induced fibrosis. Bleomycin-challenged mice were killed at days 14, 21, and 42 and their lungs were isolated for analysis. (a) Lungs were digested to generate a cellular suspension; the cells were stained with the following antibodies for flow cytometry: Gr-1, CD11b, CD11c, F4/80, and CD80. (b) Mice were unchallenged (i.e., naïve) or received bleomycin 14 or 21 days previously. Other groups of mice (*n* = 5–10/group) were treated with control IgG or anti-Gr-1 mAb at day 21 and were subsequently analyzed at day 42 after bleomycin. Whole lungs were fixed, paraffin-embedded, sectioned, and Masson's trichrome-stained to visualize ECM. Shown are representative images taken at 200x magnification (naïve, day 14, and day 21) or 40x magnification (day 42 and day 42 + αGr-1). (c) Quantitative PCR analysis of procollagen 3 and fibronectin 1 in whole lung samples. Data are mean ± SEM, *n* = 5–10/group, ^∗^ *P* ≤ 0.05 compared with IgG control group.

A time course analysis of the presence of Gr-1^+^ myeloid cells during bleomycin-induced fibrosis was undertaken. Bleomycin instillation into the lung induces an inflammatory/early fibrotic stage around day 14, a peak fibrotic response stage around day 21, and, finally, a resolution stage around day 42 after bleomycin instillation. As shown in Figure 2(a), Gr-1^+^ myeloid cells were not present in the lungs of naïve mice or mice at day 14 after bleomycin administration. However, at days 21 and 42 after bleomycin, flow cytometric analysis indicated that Gr-1^+^ myeloid cells were present in whole lung samples, with peak numbers of these cells present at day 42 (Figure 2(a)), where approximately 22% of the Gr-1^+^ myeloid cells also expressed F4/80 and the percentage of cells expressing CD80 was <6% (Figure 2(a)). It was noted that these Gr-1^+^ cells lacked myeloperoxidase (MPO) and CXCR2 expression (data not shown); both are markers of mature neutrophils.

To further explore the role of Gr-1^+^ myeloid cells in bleomycin-induced fibrosis, groups of mice at day 21 after bleomycin received an IgG control antibody or an anti-Gr-1 mAb and the lungs from these groups of mice were analyzed on day 42. Depleting Gr-1^+^ myeloid cells in this manner markedly enhanced the extent of fibrosis as revealed upon analysis of trichrome-stained lung sections (Figure 2(b)) and transcript expression of procollagen 3 and fibronectin 1 (Figure 2(c)), both of which were significantly increased in the anti-Gr-1 mAb-treated mice when compared to the IgG-treated control group. Collectively, these data suggested that Gr-1^+^CD11b^+^CD11c^−^ bone marrow-derived myeloid cells are recruited to fibrotic lungs, where these cells promote the resolution of fibrosis.

### 3.3. Adoptive Transfer of Gr-1^+^ Myeloid Cells Reduced Bleomycin-Induced Pulmonary Fibrosis

**Figure 3:**
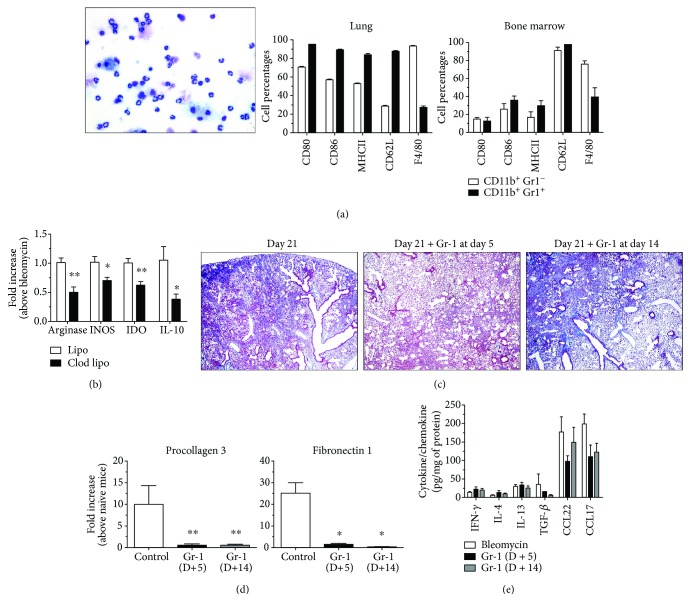
Adoptive transfer of Gr-1^+^ myeloid cells reduced lung fibrosis. Gr-1^+^ cells were purified from the lungs of bleomycin-challenged D−1 Clod Lipo-treated mice at day 5 after bleomycin. (a) Purified Gr-1^+^ myeloid cells from lung were spun onto a slide using a cytospin and stained, and a morphologic analysis was performed using light microscopy (left panel; 1000x magnification). Both lung- and bone marrow-purified CD11b^+^Gr-1^+^ and CD11b^+^Gr-1^−^ myeloid cells were stained for CD80, CD86, MHCII, CD62L, and F4/80 for flow cytometry analysis. (b) Quantitative PCR analysis of RNA isolated from Gr-1^+^ myeloid cells was used to determine transcript levels of arginase, NOS2, IDO, and IL-10. (c) Purified Gr-1^+^ myeloid cells were adoptively transferred into the lungs of bleomycin-challenged mice at days 5 (D+5) or 14 (D+14) after bleomycin. Lung samples were removed at day 21 after bleomycin. Lungs were fixed, paraffin-embedded, sectioned, and stained with Masson's trichrome. Shown are representative images taken at 100x magnification. (d) Procollagen 3 and fibronectin 1 expression were determined by quantitative PCR using RNA isolated from whole lung samples. (e) ELISA was used to determine whole lung levels of IFN-*γ*, IL-4, IL-13, TGF-*β*, CCL22, and CCL17. Data are mean ± SEM, *n* = 5–10 mice/group; ^∗^ *P* ≤ 0.05 and ^∗∗^ *P* ≤ 0.01 compared with the appropriate control group.

To further phenotype the Gr-1^+^ myeloid cells mobilized to fibrotic lungs, morphologic and flow cytometric analysis was employed on lung- and bone marrow-purified populations of these cells isolated from Clod Lipo-treated mice (D−1) at day 5 after bleomycin. Morphological analysis via light microscopy indicated that these cells were composed of a mixture of granulocytic and mononuclear cells (Figure 3(a), left panel). As previously noted, these Gr-1^+^ myeloid cells were negative for MPO and CXCR2 (data not shown). Flow cytometric analysis of lung- and bone marrow-derived CD11b^+^Gr-1^−^ and CD11b^+^Gr-1^+^ populations indicated that a higher percentage of the latter cell type stained positively for CD80, CD86, MHCII, and CD62L (Figure 3(a), middle and right panels). Whereas F4/80 was detected on >80% of CD11b^+^Gr-1^−^ myeloid cells, approximately 20% of the CD11b^+^Gr-1^+^ expressed this macrophage marker (Figure 3(a), middle and right panels).

Since CD11b and Gr-1 are both commonly expressed on cancer-associated myeloid suppressor cells (MDSC; [30, 38, 39]), lung-purified Gr-1^+^ myeloid cells from the Clod Lipo- or Lipo-treated bleomycin model at day 5 after bleomycin were analyzed for the expression of several MDSC markers. Compared with Gr-1^+^ myeloid cells isolated from mice that received bleomycin alone, Lipo treatment had no effect on the transcript expression of MDSC markers such as arginase, nitric oxide synthase-2 (NOS2), indoleamine 2,3-dioxygenase (IDO), and IL-10 but all of these markers were significantly lower in Gr-1^+^ myeloid cells from Clod Lipo-treated mice (Figure 3(b)). These data suggested that neither the Lipo alone nor the Clod Lipo treatment mobilized mature neutrophils or MDSC per se from the bone marrow. These findings also suggest that the *in vivo* Clod Lipo treatment altered the phenotype of Gr-1^+^ myeloid cells compared with the same myeloid cells purified from Lipo-treated mice.

To elucidate whether Gr-1^+^ myeloid cells are key components in the protection against and/or resolution of fibrosis, purified Gr-1^+^ myeloid cells from lungs of Clod Lipo-treated and bleomycin-challenged mice at day 5 were adoptively transferred via oropharyngeal instillation into the lungs of other groups of mice at either day 5 or 14 after bleomycin. Histological analysis of whole lungs from these mice revealed that the adoptive transfer of these cells at either day 5 or 14 resulted in a marked reduction in the extent of pulmonary fibrosis compared with untreated bleomycin-challenged lungs at day 21 (Figure 3(c), middle, right, and left panels) and a significant decrease in the expression of both procollagen 3 and fibronectin 1 transcripts in the fibrotic lungs (Figure 3(d)). Further, there were no differences in the levels of IFN-*γ*, IL-4, and IL-13 but lower levels of TGF-*β*, CCL22, and CCL17 were detected in lung samples from mice that received Gr-1^+^ myeloid cells versus those that did not (Figure 3(e)). Thus, these results suggest that mobilized bone marrow-derived Gr-1^+^ myeloid cells differ from MDSC and play a role in the resolution of bleomycin-induced pulmonary fibrosis.

### 3.4. The Coculture of Gr-1^+^ Myeloid Cells with Primary Lung Fibroblasts Decreased Fibroblast Expression of Fibronectin 1, Procollagen 1, Procollagen 3, and TGF-*β*

**Figure 4:**
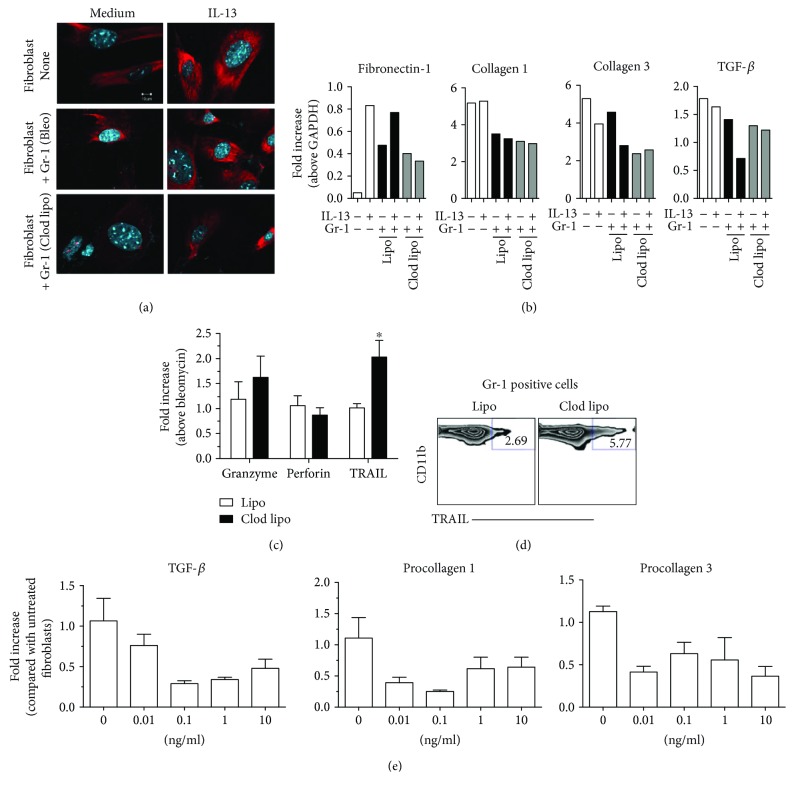
Gr-1^+^ myeloid cells decreased collagen, fibronectin, and TGF-*β* expression in cultured primary lung fibroblasts. Mice were treated with Clod Lipo or Lipo one day prior to bleomycin challenge. Five days after bleomycin administration, Gr-1^+^ myeloid cells were MACS-purified from the lungs of challenged mice, and 1 × 10^5^ Gr-1^+^ cells were cocultured with 5 × 10^5^ primary mouse lung fibroblasts for 24 h. Lung fibroblasts were either untreated or exposed to IL-13 at 10 ng/ml for 24 h and washed prior to coculture. After coculture, the Gr-1^+^ myeloid cells were subsequently washed away prior to analysis of the fibroblasts. (a) Fibroblasts were fixed, permeabilized, and stained with anticollagen mAb prior to confocal microscopy. Shown are representative images taken at 1000x magnification. (b) The expression of fibronectin 1, procollagen 1, procollagen 3, and TGF-*β* was determined using quantitative PCR analysis of adherent primary lung fibroblasts. (c) Quantitative PCR analysis of RNA was used to determine the expression of granzyme, perforin, and TRAIL in purified Gr-1^+^ myeloid cells. (d) Purified Gr-1^+^ myeloid cells were stained for CD11b, and TRAIL and analyzed by flow cytometry. (e) Purified mouse lung fibroblasts were treated with increasing concentrations of sTRAIL for 24 h, washed, and quantitative PCR analysis was used to determine the expression of TGF-*β*, procollagen 1, and procollagen 3. Data are mean ± SEM, *n* = 3–5 independent experiments. All statistics were performed using unpaired parametric *t*-tests; ^∗^ *P* ≤ 0.05.

Since the increased presence of Gr-1^+^ myeloid cells had a marked effect on the expression of ECM proteins such as procollagen 3 and fibronectin 1 and the profibrotic mediator, TGF*-β*, we hypothesized that these cells might inhibit myofibroblast ECM generation. To test this hypothesis, purified Gr-1^+^ myeloid cells were cocultured with mouse primary lung fibroblasts (at a Gr-1^+^ : fibroblast ratio of 1 : 5) for 24 h. Collagen protein expression in fibroblasts was subsequently visualized using confocal microscopy. The presence of Gr-1^+^ myeloid cells from Lipo-treated mice reduced while Gr-1^+^ myeloid cells from Clod Lipo-treated mice abolished collagen staining in fibroblasts (Figure 4(a)). Further, quantitative PCR analysis indicated that cocultured Gr-1^+^ myeloid cells decreased the transcript expression of fibronectin 1, procollagen 1, procollagen 3, and TGF-*β* in fibroblasts to a greater extent when the myeloid cells were derived from Clod Lipo-treated rather than Lipo-treated mice (Figure 4(b)). However, when Gr-1^+^ myeloid cells from Lipo- but not Clod Lipo-treated mice were cocultured in the presence of IL-13, there was a marked increase in fibronectin 1 and decrease in collagen 3 and TGF-*β* expression in fibroblasts (Figure 4(b)). Together, these results suggest that Gr-1^+^ myeloid cells might resolve pulmonary fibrosis through direct inhibitory effects on activated lung fibroblasts.

To further characterize the inhibitory effect of Gr-1^+^ myeloid cells on lung fibroblasts, RNA was analyzed from lung-purified Gr-1^+^ myeloid cells from Lipo- and Clod Lipo-treated mice at day 5 after bleomycin, and the expression of apoptosis-inducing factors was determined. Although there was no significant difference in the expression of granzyme or perforin, there was a significant increase in the expression of TRAIL in myeloid cells derived from Clod Lipo-treated when compared to Gr-1^+^ myeloid cells from Lipo-treated mice (Figure 4(c)). These transcript data were consistent with flow cytometric data indicating that the percentage of CD11b^+^Gr-1^+^TRAIL^+^ myeloid cells was higher in Clod Lipo-treated lungs when compared with Lipo-treated lungs at day 21 after bleomycin (5.77% versus 2.69%, resp.; Figure 4(d)). Thus, these data suggested that Gr-1^+^ myeloid cells might inhibit fibroblast activity in a TRAIL-dependent manner. Thus, additional *in vitro* experiments were undertaken in which mouse primary lung fibroblasts were exposed to recombinant sTRAIL (0, 0.01, 0.1, 1, or 10 ng/ml) for 24 h after which transcript analyses for TGF-*β*, procollagen 1, and procollagen 3 were performed. As shown in Figure 4(e), there was a marked dose-dependent reduction in TGF-*β*, procollagen 1, and procollagen 3 expression in response to TRAIL treatment of primary mouse fibroblasts. Thus, these results suggested that Gr-1^+^ myeloid cells might resolve bleomycin-induced fibrosis via a TRAIL-dependent mechanism.

### 3.5. TRAIL Expression Is Upregulated during the Resolution of Bleomycin-Induced Fibrosis

**Figure 5:**
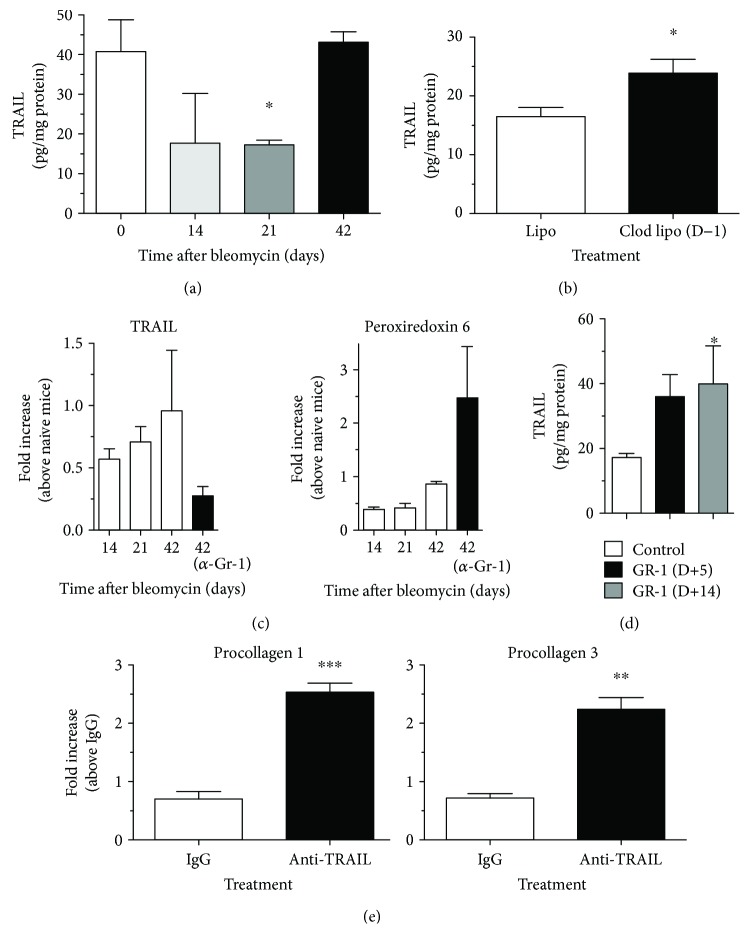
TRAIL expression is required for the optimal resolution of lung fibrosis *in vivo*. (a) Mice received bleomycin and were killed at days 14, 21, and 42 after challenge and ELISA was used to determine the concentration of TRAIL in whole lung samples. As a control, whole lung samples from naïve mice were subjected to the same analysis. (b) Mice were treated with Clod Lipo one day (D−1) prior to bleomycin, and ELISA was used to determine the whole lung levels of TRAIL. (c) Bleomycin-challenged mice were analyzed at days 14, 21, and 42 after challenge. Another group of mice were injected with IgG control or anti-Gr-1 mAb on day 21 and analyzed at day 42. Quantitative PCR analysis was used to determine TRAIL and peroxiredoxin 6 transcript expression in whole lung RNA samples. (d) Purified Gr-1^+^ myeloid cells were adoptively transferred into bleomycin-challenged mice at day 5 or 14 after bleomycin. At day 21, ELISA was used to determine TRAIL levels in all groups of mice. (e) Mice were injected by intraperitoneal injection with anti-TRAIL mAb from days 14 to 21 after bleomycin, and quantitative PCR was used to determine whole lung transcript expression of procollagen 1 and procollagen 3. Data are mean ± SEM, *n* = 5–10 mice/group, ^∗^ *P* ≤ 0.05 compared with the appropriate control group. All statistics were performed using unpaired parametric *t*-tests; ^∗∗^ *P* ≤ 0.01 and ^∗∗∗^ *P* ≤ 0.001.

To further characterize the role of TRAIL in bleomycin-induced fibrosis, TRAIL protein expression was determined in the lungs of mice at days 0, 14, 21, and 42 after bleomycin administration. TRAIL protein was significantly reduced at day 21 but levels of this protein normalized (similar to levels observed at day 0) at day 42 after bleomycin (Figure 5(a)), indicating that TRAIL expression was upregulated during the resolution of bleomycin-induced fibrosis. Further, consistent with increased Gr-1^+^ myeloid cell recruitment into the lungs, it was observed that Clod Lipo pretreatment (D−1) prior to bleomycin administration significantly increased whole lung TRAIL protein expression when compared to Lipo pretreated mice (Figure 5(b)). In addition, when mice were treated with anti-Gr-1 mAb on day 21, TRAIL mRNA expression was markedly reduced at day 42 compared with the IgG-treated group (Figure 5(c), left panel). Further, transcript levels of peroxiredoxin 6, a TRAIL inhibitor [40], were significantly increased in the lungs of mice treated with anti-Gr-1 mAb compared with the IgG-treated group (Figure 5(c), right panel). Oropharyngeal instillation of Gr-1^+^ myeloid cells at days 5 or 14 after bleomycin challenge increased TRAIL protein expression in the lungs of recipient mice at day 21 after bleomycin (Figure 5(d)). Finally, anti-TRAIL mAb treatment (via i.p. injection) from days 14 to 21 after bleomycin instillation significantly increased the expression of procollagen 1 and procollagen 3 in the lung compared with the IgG control group (Figure 5(e)). Thus, these results further suggest that Gr-1^+^TRAIL^+^ myeloid cells promote the resolution of bleomycin-induced pulmonary fibrosis.

### 3.6. Plasma TRAIL Concentration Is Increased in IPF Patients

**Figure 6:**
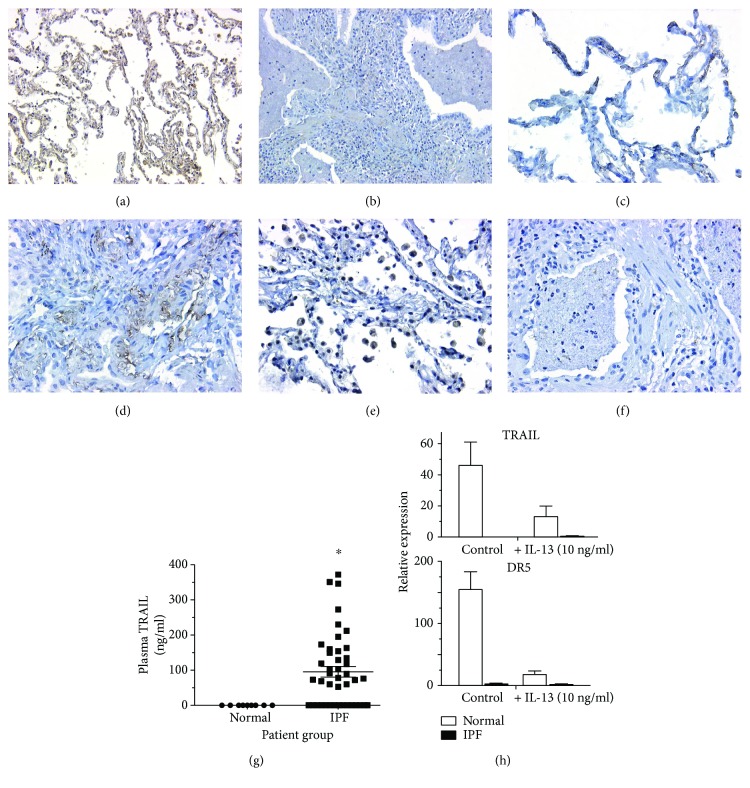
Altered TRAIL and DR5 expression in plasma, whole lung tissues, and primary fibroblasts from IPF patients. (a–f) Lung biopsies were obtained from nonfibrotic disease or IPF patients, fixed, paraffin-embedded, sectioned, and stained for TRAIL, DR5, or CD33. (a, c, e) Normal lungs stained for TRAIL, DR5, and CD33, respectively. (b, d, f) IPF lungs stained for TRAIL, DR5, and CD33, respectively. Statistical analysis were performed using ordinary one-way ANOVA test; ^∗^ *P* ≤ 0.05. (g) Plasma was collected from normal volunteers and IPF patients and ELISA was used to determine soluble TRAIL concentrations. (h) Lung fibroblasts were purified from nonfibrotic or IPF lung biopsies. Fibroblasts were left untreated or treated 10 ng/ml of IL-13 for 24 h. Quantitative PCR was used to determine the expression of TRAIL and DR5. Data are mean ± SEM, *n* = 3–5 independent experiments.

Although these results suggested an important role for TRAIL^+^ myeloid cells in the resolution of experimental bleomycin-induced pulmonary fibrosis, it was unclear whether these findings had any relevance to clinical fibrotic lung disease. Accordingly, nondiseased control and IPF lung tissues, plasma, and primary human fibroblasts were screened for the presence of TRAIL. A histological analysis is summarized in Table 1 and indicates that median TRAIL protein expression was higher in normal explanted lung tissues as compared to IPF lung biopsies (Figures 6(a) and 6(b), resp.). TRAIL-R2 or DR5 was expressed in alveolar epithelial cells and alveolar macrophages in normal lung sections (Figure 6(c)), whereas in IPF lung sections, there was focal DR5 staining in vascular-rich areas and some diffuse staining in the interstitium (Figure 6(d)). Further, normal lung sections contained numerous CD33^+^ cells but there were few of these myeloid cells in IPF lung sections (Figures 6(e) and 6(f)). There was a statistically significant increase in the concentration of sTRAIL in plasma from IPF patients (Figure 6(g)). Indeed, TRAIL was elevated but its receptors were downregulated in gene expression datasets from IPF lung biopsies and explants relative to normal lung explants (Figures S1A and S1B, resp.). Further, TRAIL receptors were abundantly expressed on microdissected hyperplastic epithelial cells adjacent to fibroblastic foci (Figure S2A) but not in fibroblastic foci (Figure S2B) relative to normal nondiseased regions of the lungs. Finally, gene expression arrays of BAL cells from two different cohorts of IPF patients showed a loss of TRAIL transcript expression relative to normal donor BAL (Figures S3A and S3B). TRAIL and DR5 transcript expression was largely absent from primary IPF fibroblasts compared with primary normal fibroblasts (Figure 6(h)). One explanation for the loss of TRAIL and DR5 in IPF fibroblasts might relate to the IL-13-enriched environment from which they were derived [37]. Indeed, the expression of both TRAIL and its receptor was strongly decreased in normal lung fibroblasts exposed to IL-13 for 24 h (Figure 6(h)). Together, these findings suggest that although TRAIL is present in soluble form in IPF, there is a lack of TRAIL^+^ myeloid cells in IPF lung samples and IPF fibroblasts lack DR5 expression, presumably due, in part, to the inhibitory effects of IL-13.

## 4. Discussion

The present study addressed a novel mechanism through which bleomycin-induced pulmonary fibrosis is resolved in the mouse. Bleomycin-induced lung injury is widely used to explore antifibrotic therapeutics despite the fact that mice surviving an intrapulmonary bleomycin challenge normally resolve the majority of the ensuing fibrosis [41]. We addressed the mechanism leading to the resolution of fibrosis in this model via the exploration of the presence and role of bone marrow-derived Gr-1^+^ myeloid cells. To assess the role of bone marrow derived Gr-1^+^ myeloid cells in lung fibrosis, clodronate-loaded liposomes were intraperitoneally administered into mice. Intraperitoneal administration of Clod Lipo has been previously observed to deplete bone marrow, spleen, liver, blood, lymph node, and peritoneal macrophages and dendritic cells (http://clodronateliposomes.org/). In our studies, we utilized a liposome dose of 0.1 ml/20 g of body weight, which is approximately half the dose recommended by the manufacturer for depletion of macrophages and dendritic cells. With this dose, there were no changes in lung-associated F4/80^+^ macrophages or CD11b^+^CD11c^+^ cells; however, there was a significant elevation of Gr-1^+^CD11c^−^ myeloid cells in the lungs of the Clod Lipo-treated groups. Given known role of clodronate in depleting macrophages, we hypothesized that elevated Gr-1^+^ cells in the Clod Lipo treated groups was a consequence of macrophage depletion. Indeed, CD11c^+^ cell depletion in mouse bone marrow induces mobilization and increased pulmonary presence of Ly6G (a marker that is also detected with anti-Gr-1 antibodies) expressing cells in mice [42]. These results suggest that macrophage/dendritic cell depletion via Clod Lipo treatment in our model might induce the mobilization and increased lung recruitment of bone marrow resident Gr-1^+^ cells.

Using clodronate liposome-based, antibody-based, and cell adoptive transfer approaches, we observed that Gr-1^+^ myeloid cells exerted a prominent effect on the resolution of pulmonary fibrosis. These cells resolved pulmonary fibrosis via a TRAIL-dependent mechanism as revealed both *in vitro* and *in vivo*. TRAIL was present in both IPF plasma and biopsies, and its transcript was elevated in both IPF lung biopsies and explants but lost in IPF BAL cells; however, transcripts for its major receptors were downregulated in both IPF lung biopsies and explants and protein levels for one of its major receptors, DR5, was largely absent from both IPF biopsies and primary IPF fibroblasts. Finally, IL-13 had a major inhibitory effect on DR5 expression by normal human fibroblasts. Taken together, TRAIL-mediated resolution of experimental pulmonary fibrosis represents an attractive therapeutic approach that requires further investigation in clinical fibrotic lung disease.

Nonlethal doses of bleomycin sulfate typically induce an early inflammatory response in the lung that peaks around the first week after challenge, which is followed by a fibrotic phase that peaks around day 21 [43]. Fibrosis does not persist in this model and major resolution of this pathologic feature is typically observed around 42 days after bleomycin challenge. We speculated that a bone marrow-derived cell might be involved in this resolution process and initially employed clodronate liposomes to explore this hypothesis. Clodronate has been recently shown to induce the apoptosis of bone marrow-resident macrophages thereby leading to the release of hematopoietic and mesenchymal stem cells from the bone marrow [32, 33]. This was of interest to us since other earlier reports indicated that approaches leading to the mobilization of bone marrow cells (including but not limited to hematopoietic stem cells, endothelial, and epithelial progenitor cells) reduced bleomycin-induced lung fibrosis [20, 21, 44] whereas the ablation of bone marrow cells increased the fibrotic response to bleomycin in mice [18]. Further, bone marrow-derived mesenchymal stem cells have been shown to play a protective role against bleomycin and autoimmune-induced pulmonary fibrosis in mice [18, 25]. In the present study, a Gr-1^+^ myeloid cells were identified in the resolving bleomycin-challenged lung that did not match the profile of mature neutrophils or MDSCs. MDSCs have potent immunomodulatory properties due to their expression and activity of arginase, NOS2, IDO, NADPH, IL-10, and prostaglandin [29, 30, 38, 39, 45, 46]. In this bleomycin model, neither liposome treatment mobilized MDSCs, and the Gr-1^+^ cells purified from Clod Lipo-treated, bleomycin-challenged mice exhibited significantly less arginase, NOS2, IDO, and IL-10 expression compared with the same cells purified from Lipo-treated, bleomycin-challenged mice. There were more Gr-1^+^ myeloid cells in fibrotic lungs of Clod Lipo-treated compared with Lipo-treated mice, which we hypothesize was due to the enrichment of these cells in the former group due to the loss of the Gr-1^+^ F4/80^+^ population. Morphologically, these Clod Lipo-treated Gr-1^+^ cells resembled bone marrow myeloid cells most similar to a population of myeloid cells reported to be recruited into the lungs of LPS-challenged asthmatic mice [29]. However, further investigation to confirm the identity of these cells is required. Thus, the bone marrow is the source of unique myeloid cells with both antifibrotic and tissue repairing properties.

The manner in which Gr-1^+^ cells attenuated bleomycin-induced pulmonary fibrosis was a major focus of the present study. While changes in profibrotic cytokine and chemokine levels such as IL-13 [47], CCL17, and CCL22 [43] were observed in the lungs of mice treated with Clod Lipo or adoptively transferred with Gr-1^+^ cells, many of these changes were not consistent between our Clod Lipo-treated groups and/or did not reach statistical significance. However, when mice were treated with Clod Lipo or received purified Gr-1^+^ cells, there was a consistent reduction in the expression of ECM proteins in fibrotic lungs suggesting that Gr-1^+^ cells might inhibit myofibroblast activation and ECM protein expression. This was confirmed *in vitro* by coculturing purified Clod Lipo induced Gr-1^+^ cells with primary lung fibroblasts. In these studies, we utilized a Gr-1^+^ cell to a fibroblast ratio of 1 : 5. Despite the low relative numbers of Gr-1^+^ myeloid cells in our coculture studies, we observed that Gr-1^+^ cells inhibited the expression of collagen protein and other ECM markers such as fibronectin, procollagen, and TGF-*β* by cultured fibroblasts. These coculture experiments also suggested that the Gr-1^+^ cells were promoting the death and/or inhibiting the viability of cultured fibroblasts. It is possible that these effects may be more prominent with higher numbers of myeloid cells, and future studies are warranted to better characterize the effects of these Gr-1^+^ cells on lung fibroblasts.

In a normal wound healing response, the inhibition of fibrosis is typically achieved by the induction of myofibroblast apoptosis [48]. A molecular survey of known cell membrane-associated proapoptotic factors revealed that TRAIL was prominently expressed on this Gr-1^+^ myeloid cell population. TRAIL is a well-known mediator of apoptotic cell death that is expressed by a number of bone marrow-derived cells including neutrophils [49]. Recent evidence suggest that TRAIL-induced apoptosis is involved in the reduction of bleomycin-induced lung fibrosis as evidenced by the significant decrease in TUNEL^+^ cells in the lungs of TRAIL^−/−^ mice compared with TRAIL^+/+^ mice at day 23 after bleomycin challenge [15]. Further, this protein has been recently shown to play a prominent role in the resolution of hepatic fibrosis and amelioration of cirrhosis [50–54]. Herein, the greatest levels of TRAIL mRNA and protein were detected in lung samples during the resolution phase in this bleomycin model, a phase in which mature neutrophils are typically absent from the lung. This change in TRAIL levels correlated with the increased recruitment of Gr-1^+^ myeloid cells into fibrotic lungs, which peaked at day 42 after bleomycin instillation, suggesting that these cells might be the primary source of TRAIL in the fibrotic lungs. Interestingly, there was a marked increase in the expression of peroxiredoxin 6, a TRAIL inhibitor [40], in mice treated with anti-Gr-1 mAb prior to bleomycin suggesting that in addition to the expression of the proapoptotic receptor TRAIL, Gr-1^+^ myeloid cells might also downregulate TRAIL inhibitors during the resolution of bleomycin induced fibrosis.

An analysis of human plasma, lung tissues, and primary fibroblasts revealed that our experimental findings might have important therapeutic utility in the clinic. While our plasma data differ from the serum data generated by McGrath et al. [15] who observed a significant decrease in TRAIL concentration in the serum from IPF patients when compared to normal subjects, the results from both studies suggest that there might be a lack of TRAIL and TRAIL-mediated effects in the lungs of IPF patients. This is supported by a report examining the expression of TRAIL and its receptors in normal and IPF lungs, where both TRAIL protein and its receptors were expressed by epithelial cells adjacent to fibroblastic foci and, to a lesser extent, in fibroblastic foci; however, unlike hyperplastic epithelial cells, fibroblasts present in the foci were devoid of apoptosis inducing p53 protein [55].

The lack of responsiveness of fibroblasts to TRAIL might be due to the lack of TRAIL ligand (as a consequence of decreased expression and/or myeloid cell recruitment into the lungs, as revealed by decreased CD33 cells), internalization of the TRAIL receptors, and increased presence of IL-13 or other intrinsic differences between IPF and normal lung fibroblasts. Our results suggest that a combination of these effects may be in play. Basally, normal but not IPF lung fibroblasts express sufficient levels of TRAIL and DR5 transcripts and are sensitive to TRAIL-mediated apoptosis. Further, IL13 stimulation markedly reduced both proteins in normal lung fibroblasts. Despite elevated expression of the IL13 receptors, IL13R*α*1, and IL13R*α*2, in IPF relative to normal lung fibroblasts [37], transcriptomic analysis suggested that IL13 is not expressed in IPF lung fibroblast cultures (not shown). Collectively, these results suggest that the lack of TRAIL and DR5 expression in IPF lung fibroblasts might be due to other mechanisms in addition to IL13 signaling. Indeed, experimental data in our laboratory suggest that resistance to TRAIL-mediated apoptosis by IPF lung fibroblasts may in part be due to the senescent state of these cells (data not shown). However, given that there was an effect of IL13 stimulation on the expression of TRAIL and its receptor, DR5, on nondiseased fibroblasts and the elevated expression of IL13 mRNA in IPF relative to normal lungs [56], targeting IL-13 signaling might help sensitize a subset of lung fibroblasts to the effects of TRAIL in IPF patients.

In summary, we demonstrate that Gr-1^+^TRAIL^+^ myeloid cells promote the resolution of experimental pulmonary fibrosis. Clinically targeting TRAIL-resistant cancer cells has been problematic because these cells express several apoptotic inhibitors and prosurvival signals such as c-FLIP and PI3K/Akt pathway activation. Unfortunately, IPF myofibroblasts express many of these factors and aberrant pathways [9–11]. Based on the present studies, TRAIL remains an attractive therapeutic target in IPF after strategies are identified that sensitize myofibroblasts to TRAIL-induced inhibitory effects.

## Figures and Tables

**Table 1:** Human lung biopsy histological analysis summary.

Disease and sample ID	TRAIL	DR5	CD33
*Control*			
NL#8	++	+	+
NL#9	++	+	+
*IPF*			
01K236	++	−	−
00K1258	++	−	−
01K1197	+/−	+	+/−
03K796	ND	+	ND
01K2387	+/−	+	−

ND: not detected; +/−: patchy staining; +: positive staining; ++: strong staining; −: no staining.

## Supplementary Materials

Supplementary 1 Figure S1: expression of apoptotic mediators in diagnostic IPF lung biopsies and end-stage IPF lung explants. Publicly available gene expression datasets (GSE24206) were mined from NCBI's geo datasets database. Gene expression values were extracted for IPF lung biopsies versus normal lungs (left) and IPF lung explants versus normal lungs (right) using NCBI's Geo2R gene expression analysis tool, and the expression data were uploaded onto ingenuity IPA. Shown is a modified version of Ingenuity's Apoptosis canonical pathway, overlaid with the GSE24206 gene expression fold changes (bottom) and *P* values (top). Red—upregulated transcripts by ≥1.5-fold and a *P* value ≤ 0.05; green—downregulated transcripts by ≥1.5-fold and a *P* value ≤ 0.05.

Supplementary 2 Figure S2: expression of apoptotic mediators in microdissected IPF epithelial cells and fibroblastic foci. Publicly available gene expression datasets (GSE35309) were mined from NCBI's geo datasets database. Gene expression values were extracted for hyperplastic epithelial cells adjacent to fibroblastic foci versus normal tissue (left) and fibroblastic foci versus normal lung tissue (right) using NCBI's Geo2R gene expression analysis tool, and the expression data were uploaded onto ingenuity IPA. Shown is a modified version of Ingenuity's Apoptosis canonical pathway, overlaid with the GSE35309 gene expression fold changes (bottom) and *P* values (top). Red—upregulated transcripts by ≥1.5-fold and a *P* value ≤ 0.05; green—downregulated transcripts by ≥1.5-fold and a *P* value ≤ 0.05.

Supplementary 3 Figure S3: expression of apoptotic mediators in IPF BAL cells. Publicly available gene expression datasets (GSE70867) were mined from NCBI's geo datasets database. Gene expression values were extracted for IPF BAL Siena cohort (A) and Freiburg cohort (B) versus normal donor BAL using NCBI's Geo2R gene expression analysis tool, and the expression data were uploaded onto ingenuity IPA. Shown is a modified version of Ingenuity's Apoptosis canonical pathway, overlaid with the GSE70867 gene expression fold changes (bottom) and *P* values (top). Red—upregulated transcripts by ≥1.5-fold and a *P* value ≤ 0.05; green—downregulated transcripts by ≥1.5-fold and a *P* value ≤ 0.05.
