# Supplementary figures and images for: TRAIL-Dependent Resolution of Pulmonary Fibrosis
Source: Mediators Inflamm. 2018 Jan 24;2018:7934362. doi: 10.1155/2018/7934362 (PMC5833466; doi:10.1155/2018/7934362)

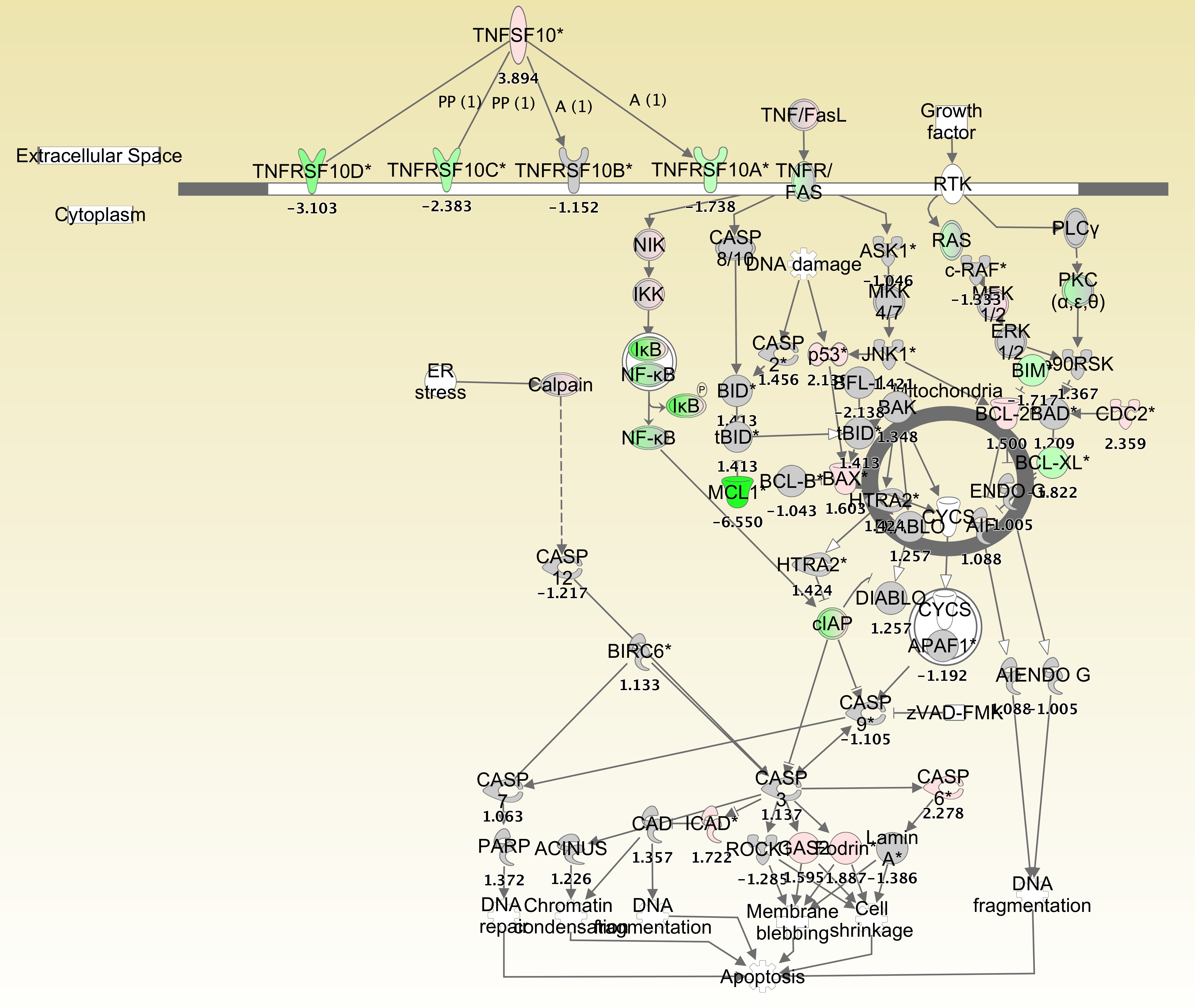

Supplement: Supplementary 1 — Figure S1: expression of apoptotic mediators in diagnostic IPF lung biopsies and end-stage IPF lung explants. Publicly available gene expression datasets (GSE24206) were mined from NCBI's geo datasets database. Gene expression values were extracted for IPF lung biopsies versus normal lungs (left) and IPF lung explants versus normal lungs (right) using NCBI's Geo2R gene expression analysis tool, and the expression data were uploaded onto ingenuity IPA. Shown is a modified version of Ingenuity's Apoptosis canonical pathway, overlaid with the GSE24206 gene expression fold changes (bottom) and P values (top). Red—upregulated transcripts by ≥1.5-fold and a P value ≤ 0.05; green—downregulated transcripts by ≥1.5-fold and a P value ≤ 0.05. [file 7934362.f1.zip › 7934362.f1/Fig S1a_MI_2085399.jpg]

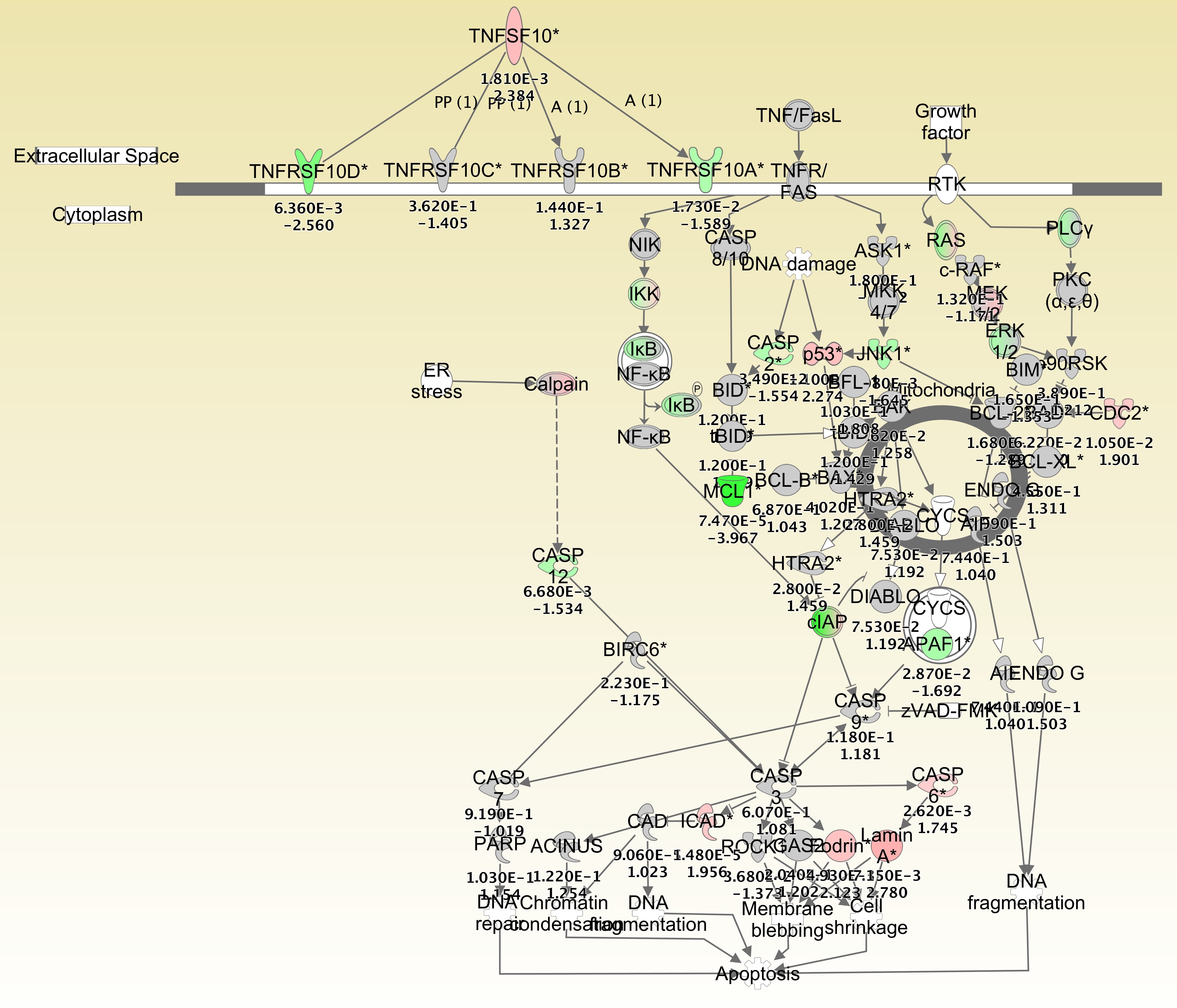

Supplement: Supplementary 1 — Figure S1: expression of apoptotic mediators in diagnostic IPF lung biopsies and end-stage IPF lung explants. Publicly available gene expression datasets (GSE24206) were mined from NCBI's geo datasets database. Gene expression values were extracted for IPF lung biopsies versus normal lungs (left) and IPF lung explants versus normal lungs (right) using NCBI's Geo2R gene expression analysis tool, and the expression data were uploaded onto ingenuity IPA. Shown is a modified version of Ingenuity's Apoptosis canonical pathway, overlaid with the GSE24206 gene expression fold changes (bottom) and P values (top). Red—upregulated transcripts by ≥1.5-fold and a P value ≤ 0.05; green—downregulated transcripts by ≥1.5-fold and a P value ≤ 0.05. [file 7934362.f1.zip › 7934362.f1/Fig S1b_MI_2085400.jpg]

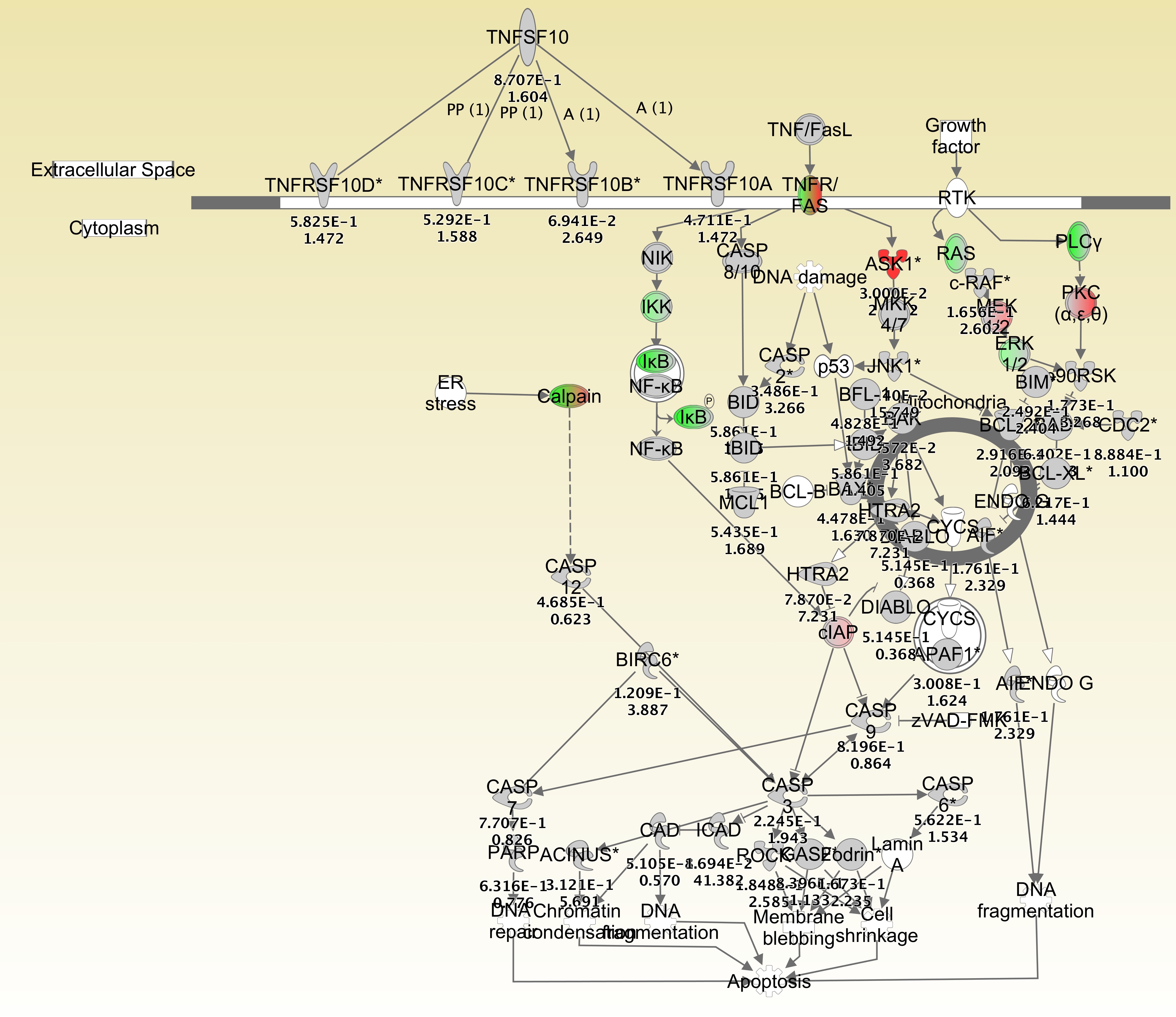

Supplement: Supplementary 2 — Figure S2: expression of apoptotic mediators in microdissected IPF epithelial cells and fibroblastic foci. Publicly available gene expression datasets (GSE35309) were mined from NCBI's geo datasets database. Gene expression values were extracted for hyperplastic epithelial cells adjacent to fibroblastic foci versus normal tissue (left) and fibroblastic foci versus normal lung tissue (right) using NCBI's Geo2R gene expression analysis tool, and the expression data were uploaded onto ingenuity IPA. Shown is a modified version of Ingenuity's Apoptosis canonical pathway, overlaid with the GSE35309 gene expression fold changes (bottom) and P values (top). Red—upregulated transcripts by ≥1.5-fold and a P value ≤ 0.05; green—downregulated transcripts by ≥1.5-fold and a P value ≤ 0.05. [file 7934362.f2.zip › 7934362.f2/Fig S2a_MI_2085401.jpg]

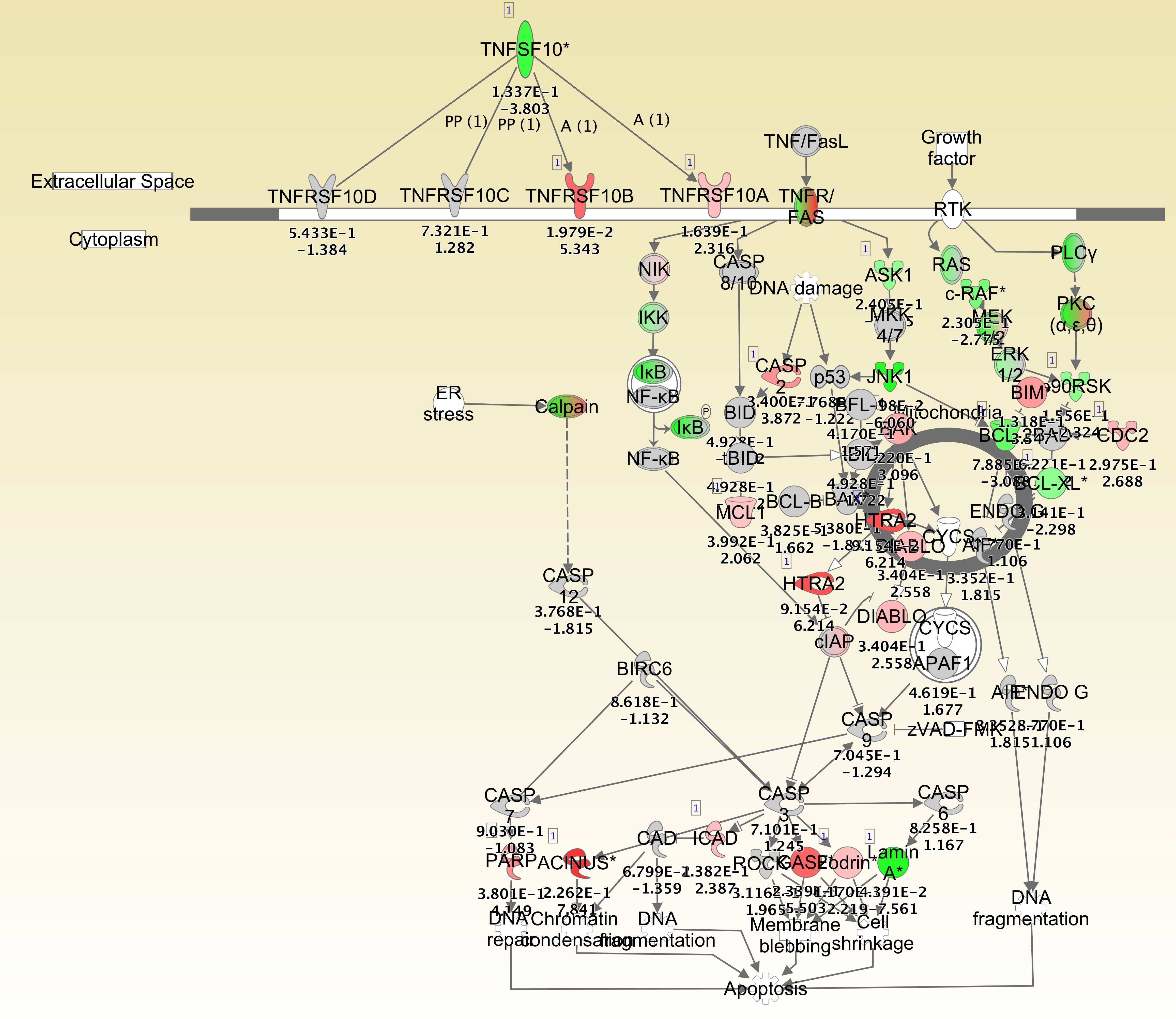

Supplement: Supplementary 2 — Figure S2: expression of apoptotic mediators in microdissected IPF epithelial cells and fibroblastic foci. Publicly available gene expression datasets (GSE35309) were mined from NCBI's geo datasets database. Gene expression values were extracted for hyperplastic epithelial cells adjacent to fibroblastic foci versus normal tissue (left) and fibroblastic foci versus normal lung tissue (right) using NCBI's Geo2R gene expression analysis tool, and the expression data were uploaded onto ingenuity IPA. Shown is a modified version of Ingenuity's Apoptosis canonical pathway, overlaid with the GSE35309 gene expression fold changes (bottom) and P values (top). Red—upregulated transcripts by ≥1.5-fold and a P value ≤ 0.05; green—downregulated transcripts by ≥1.5-fold and a P value ≤ 0.05. [file 7934362.f2.zip › 7934362.f2/Fig S2b_MI_2085402.jpg]

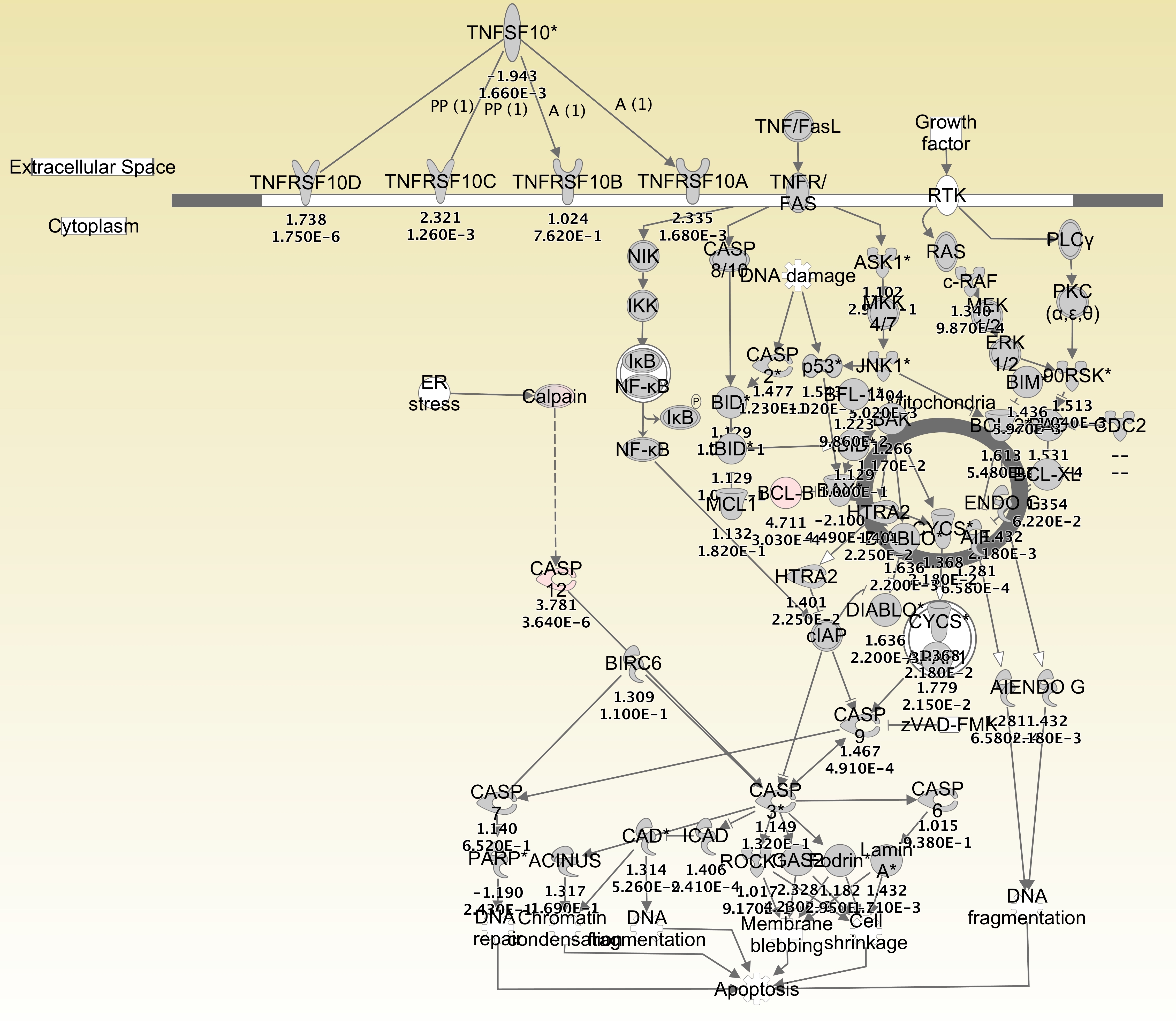

Supplement: Supplementary 3 — Figure S3: expression of apoptotic mediators in IPF BAL cells. Publicly available gene expression datasets (GSE70867) were mined from NCBI's geo datasets database. Gene expression values were extracted for IPF BAL Siena cohort (A) and Freiburg cohort (B) versus normal donor BAL using NCBI's Geo2R gene expression analysis tool, and the expression data were uploaded onto ingenuity IPA. Shown is a modified version of Ingenuity's Apoptosis canonical pathway, overlaid with the GSE70867 gene expression fold changes (bottom) and P values (top). Red—upregulated transcripts by ≥1.5-fold and a P value ≤ 0.05; green—downregulated transcripts by ≥1.5-fold and a P value ≤ 0.05. [file 7934362.f3.zip › 7934362.f3/Fig S3a_MI_2085403.jpg]

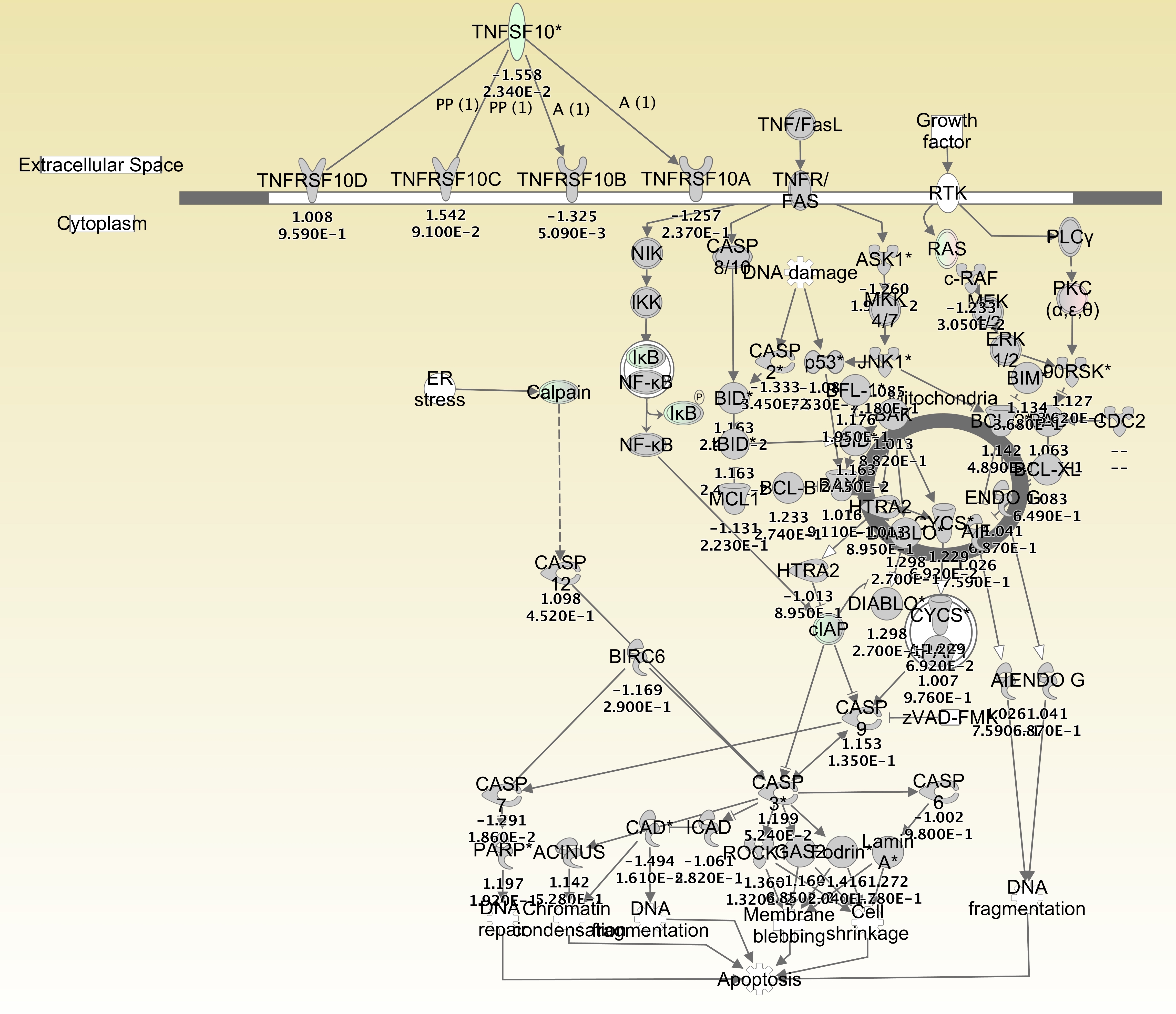

Supplement: Supplementary 3 — Figure S3: expression of apoptotic mediators in IPF BAL cells. Publicly available gene expression datasets (GSE70867) were mined from NCBI's geo datasets database. Gene expression values were extracted for IPF BAL Siena cohort (A) and Freiburg cohort (B) versus normal donor BAL using NCBI's Geo2R gene expression analysis tool, and the expression data were uploaded onto ingenuity IPA. Shown is a modified version of Ingenuity's Apoptosis canonical pathway, overlaid with the GSE70867 gene expression fold changes (bottom) and P values (top). Red—upregulated transcripts by ≥1.5-fold and a P value ≤ 0.05; green—downregulated transcripts by ≥1.5-fold and a P value ≤ 0.05. [file 7934362.f3.zip › 7934362.f3/Fig S3b_MI_2085404.jpg]
